# No compromise between metabolism and behavior of decorator crabs in reduced pH conditions

**DOI:** 10.1038/s41598-019-42696-8

**Published:** 2019-04-18

**Authors:** Ashley Rankin, Kyungah Seo, Olivia A. Graeve, Jennifer R. A. Taylor

**Affiliations:** 1 Scripps Institution of Oceanography, Marine Biology Research Division, University of California, San Diego, La Jolla, CA 92093 USA; 2 Department of Mechanical and Aerospace Engineering, University of California, San Diego, La Jolla, CA 92093-0411 USA

**Keywords:** Animal behaviour, Animal physiology

## Abstract

Many marine calcifiers experience metabolic costs when exposed to experimental ocean acidification conditions, potentially limiting the energy available to support regulatory processes and behaviors. Decorator crabs expend energy on decoration camouflage and may face acute trade-offs under environmental stress. We hypothesized that under reduced pH conditions, decorator crabs will be energy limited and allocate energy towards growth and calcification at the expense of decoration behavior. Decorator crabs, *Pelia tumida*, were exposed to ambient (8.01) and reduced (7.74) pH conditions for five weeks. Half of the animals in each treatment were given sponge to decorate with. Animals were analyzed for changes in body mass, exoskeleton mineral content (Ca and Mg), organic content (a proxy for metabolism), and decoration behavior (sponge mass and percent cover). Overall, decorator crabs showed no signs of energy limitation under reduced pH conditions. Exoskeleton mineral content, body mass, and organic content of crabs remained the same across pH and decoration treatments, with no effect of reduced pH on decoration behavior. Despite being a relatively inactive, osmoconforming species, *Pelia tumida* is able to maintain multiple regulatory processes and behavior when exposed to environmental pH stress, which underscores the complexity of responses within Crustacea to ocean acidification conditions.

## Introduction

As the world’s oceans experience unparalleled rates of atmospheric carbon dioxide input (i.e., ocean acidification), it is becoming increasingly understood that the potential impacts on marine animals are far reaching and generally negative^1,2^. Many marine calcifiers experience sub-lethal stress leading to metabolic depression^3^, reduced growth rate^4^, reduced energy storage ability^5^, and changes in calcification^3^ under reduced pH conditions associated with ocean acidification. Within crustaceans, the effects are highly variable and the data are still too limited to resolve general patterns, if they exist. Consider that calcification, one of the most commonly measured responses, decreases under reduced pH conditions for some crustacean species and life stages^6–8^, yet increases in others^9–11^, or remains unchanged^1,12–16^. The root of this variability remains unclear, though current hypotheses suggest that the most vulnerable species may be those that are poor osmoregulators, are inactive with low metabolisms, and inhabit stable environments^1,17,18^. The dynamic intertidal environment, however, does not absolve species from susceptibility^12,13,19–21^.

In general, stressful conditions can increase the metabolic activity of animals as their body compensates for changes in the environment and attempts to maintain homeostasis^22^. For crustaceans that have weak regulatory capacity, or are osmoconformers, reductions in extracellular pH in response to environmental hypercapnia may be compensated for by exoskeleton dissolution, as occurs in *Necora puber* and *Cancer productus*^23,24^. Both the extra minerals (Mg) in the hemolymph and reduced extracellular pH can lead to metabolic depression^25^, ultimately affecting growth and other processes. Even for crustacean species with strong acid-base regulatory mechanisms and osmoregulation abilities, the energetic costs of these processes may increase when exposed to ocean acidification conditions. It is a common argument that increased energy demand to cope with a stressful environment may leave animals, both osmoregulators and osmoconformers, with insufficient energy to perform all of their normal physiological processes and behaviors, resulting in trade-offs in energy allocation. This is evident in species that experience reduced growth rates^6^, changes in calcification^8^, and reduced swimming performance^26^ under reduced pH conditions. The energy and metabolic costs of responding to reduced pH has been studied in some species^20^, but typically not in combination with other physiological processes and behaviors that may be compromised. It is informative to study multiple facets of the response because vulnerability of crustaceans to ocean acidification could materialize at the physiological, morphological, and behavioral levels to affect ecological interactions and survival.

Decorator crabs (family Majidae) are interesting candidates to study energy and trade-offs under ocean acidification conditions because of their vulnerability traits and iconic camouflage behavior. Many species fit the predictions of vulnerability, whereby they are either osmoconformers or poor osmoregulators^27^ and they are typically slow-moving, relatively inactive scavengers rather than predators^28^. Distributed across intertidal and subtidal habitats, decorator crabs experience either dynamic or relatively stable ocean conditions. Perhaps their most compelling and germane feature though is that decoration camouflage is an energetically expensive, ecologically significant behavior that is easily tractable.

Decoration camouflage, in which animals attach material (organisms or debris) from the environment to their bodies, is not limited to decorator crabs; it is used by nearly 25% of metazoan phyla^29^ and is an effective strategy for avoiding predation and increasing survival^30^. Decoration behavior is considered to be energetically expensive to maintain, requiring that animals expend energy to find and manipulate decorations, as well as place and carry these decorations on their bodies^29,31,32^. Augmenting the costs of active decorating are the costs that come with developing the morphology for decoration attachment, such as setae and adhesives^28^. Indeed, many species of decorator crabs have evolved an ontogenetic shift to lower setal density (fewer setae) and decreased decoration rates as the animal grows larger^32,33^. The trend for decoration behavior to become reduced or absent as animals grow larger is commonly attributed to its energetic costs and trade-offs^28,32,34^. Though the energetic cost of decoration behavior has long been inferred and even modeled^29^, it has rarely been quantified. Berke and Woodin^32^ demonstrated in a series of starvation experiments that decorated *Oregonia gracilis* crabs had consistently greater tissue catabolism, and thus higher metabolic activity, than undecorated crabs; within just a few days of starvation, decorated crabs lost 6 to 7-fold more mass and had 8% less organic content compared to crabs without decoration. This suggests that carrying decorations results in greater energetic costs. Carrying additional load is associated with increased metabolic costs, as demonstrated in a range of other animals that carry objects as well. Slow-walking hermit crabs use twice as much oxygen when carrying mollusk shells^35^ and ants carrying food increase CO_2_ production rates by 30–60% depending on the size of the load^36,37^. For male fiddler crabs, carrying the major claw increases respiration rates by 8%^38^.

Decoration behavior may be too expensive for some animals to maintain during times of stress, when homeostatic demands are high and energy resources limited. For example, under hypoxic stress both sea urchins and decorator crabs have been observed to discard their decorations^39^. Similarly, decoration behavior is negatively affected in sea urchins exposed to elevated temperature. An increase of 3 °C results in significantly delayed decoration behavior, fewer urchins that decorate, and less shells used for cover^40^. A 4 °C increase above ambient summer temperature causes the sea urchin *Lytechinus variegatus* to stop decorating entirely^41^. If this sensitivity of decoration behavior to animal stress extends to ocean acidification, then decoration behavior and survival may be compromised for a variety of species under future ocean conditions.

Given the energetic expense of decoration behavior and the potential metabolic costs of responding to ocean acidification, we hypothesized that decorator crabs would face a trade-off between growth, calcification, and decoration behavior due to energy limitations under reduced pH conditions. We predicted that decorated crabs would have higher energetic costs than undecorated crabs, and that crabs in reduced pH would have higher energetic costs than those in ambient pH, resulting in decreased decoration behavior. We tested our hypothesis and predictions using the decorator crab, *Pelia tumida*, also known as the dwarf teardrop crab. This species inhabits the coasts of California and Mexico, from the low intertidal zone to subtidal waters as deep as 100 m^42^. It lacks an ontogenetic shift in decoration behavior, most likely due to its small size (adult carapace width: 10–15 mm^32^). Here we exposed *P*. *tumida* to ambient and reduced pH conditions for five weeks, after which change in mass, exoskeleton calcification, organic content, and decoration behavior were analyzed to determine the effects of reduced pH on animal physiology, morphology, and behavior.

## Materials and Methods

### Animal collection and maintenance

Forty-eight decorator crabs (31 male, 17 non-ovigerous female) of the species *Pelia tumida* were collected subtidally from the pier pilings of Scripps Pier, Scripps Institution of Oceanography (SIO), San Diego, California (32°52′N, 117°15′W) over the course of 3 months. Once all crabs were collected, they were maintained for 2 weeks under the same conditions prior to the start of the experiment. Crabs were housed in the Hubbs Hall experimental aquarium in individual tanks receiving flow-through seawater until the start of the experiment in July 2016. Prior to and throughout the experiment, crabs were fed an equal diet of frozen tilapia pieces (cut to 1 cm × 3 cm in size) 3 times a week, which was enough to satiate the crabs. Excess food was removed after 24 hours.

### Experimental design and maintenance

The experimental OA system consisted of two large header tanks (60.5 L) that each received filtered seawater pumped in from the SIO Pier (3–4 m depth, 300 m offshore) at ambient pH_SWS_ (8.01 ± 0.03), pCO_2_ (613 ± 109 μatm), temperature (19.1 ± 2.4 °C), and salinity (33.5 ± 0.1) (mean ± s.d. during the experimental period). One header tank was maintained at ambient pH while the second header tank was adjusted for reduced pH_SWS_ (7.74 ± 0.02). Target pH was selected based on current predictions for decreased ocean surface pH of 0.3–0.4 pH units by the year 2100^43^. Reduced pH was accomplished by bubbling in 100% CO_2_ into the treatment header tank, which was controlled by an Apex Lite aquarium controller (pH accuracy 0.01; Neptune Systems, Morgan Hill, California, United States). Both pH and temperature of the header tanks were continuously monitored with the Apex controller and data logged every 20 minutes.

Each header tank supplied flow-through seawater via emitters with individual tubes to each of 24 experimental plastic cups (0.95 L) at a rate of 38 L hr^−1^. Cups were placed randomly on a shelf below the header tanks. Each experimental cup housed an individual crab, for a combined total of 48 individuals. A small inert rock was placed in each cup for crabs to crawl on. Crabs were weighed and measured (carapace width) and then assigned to each treatment so that both duration in the lab and body size were evenly distributed across treatments (Ambient pH/undecorated: 0.54 ± 0.48 g, 8.51 ± 2.41 mm, 5 male, 7 female; Ambient pH/decorated: 0.38 ± 0.31 g, 7.97 ± 1.60 mm, 6 male, 6 female; Reduced pH/undecorated: 0.86 ± 0.65 g, 10.01 ± 2.99 mm, 10 male, 2 female; Reduced pH/decorated: 0.64 ± 0.42 g, 9.23 ± 2.20 mm, 10 male, 2 female). Both crab mass and carapace width were the same across all four treatments (Kruskall-Wallace test: mass, *H*12 = 4.586, *P* = 0.21; carapace width, *H*12 = 3.583, *P* = 0.31). Experimental pH was gradually adjusted over the course of 3 days in an attempt to minimize stress. Once the target pH was reached, the experiment was run for 5 weeks. Crabs were checked for molts and deaths daily, with exuviae promptly removed.

### Water chemistry

Daily readings of pH and temperature were taken from each header tank and experimental cup using a portable probe (HQ40d, probe PHC201, accuracy 0.01 pH, 0.01 °C temperature, Hach, Loveland, Colorado, United States). All pH probes were calibrated weekly using NBS buffer solutions. Water samples were also taken from each header tank and a subset of experimental cups at the beginning (1 random cup from each pH treatment) and end (2 random cups from each pH treatment) of the experiment in accordance with standard operating procedures^44^. Water samples were submitted to the Dickson laboratory at SIO for analysis of pH_SWS_, density-based salinity, and total alkalinity (TA) at 25 °C (Table 1). Carbonate and aragonite saturation states, concentrations of carbonate and bicarbonate, and pCO_2_ were calculated using CO_2_ Sys 2.01 (Table 1). For calculations, dissociation constants of K_1_ and K_2_ were from Mehrbach^45^, refit by Dickson and Millero^46^. The HSO_4_ constant was from Dickson^47^, the [B}_T_ value was from Uppstrom^48^, and the seawater pH scale was used. Water samples were used to calculate the average difference between the Hach pH probe readings and spectrophotometric pH values per sampling set. The mean pH offset was then used to correct daily measures of pH from the Hach probe (35 total per cup), which were then averaged for all experimental cups in each treatment (Table 1).

**Table 1:** Water temperature and carbonate chemistry parameters [mean (s.d.)] throughout the 5 week experiment.

Treatment	pCO_2_ (µatm)	pH_sws*_	Temperature* (°C)	Salinity*	TA* (µmol/kgSW)	HCO_3_ (µmol kg^−1^)	CO_3_ (µmol kg^−1^)	Ω Ca	Ω Ar
Ambient pH	613 (109)	8.01 (0.03)	21.8 (1.2)	33.5 (0.1)	2226 (10)	1915 (77)	126 (28)	3.04 (0.69)	1.97 (0.46)
Reduced pH	894 (66)	7.74 (0.02)	21.8 (1.2)	33.5 (0.1)	2226 (8)	2000 (37)	92 (11)	2.21 (0.28)	1.43 (0.19)

A total of 35 measurements were taken per experimental cup and the mean pH and temperature values for each cup were then used to calculate the overall means and standard deviations for each treatment. Measured parameters (*n* = 5 per treatment) are indicated by asterisk. All other parameters were calculated using CO_2_SYS.

### Decoration behavior

Immediately prior to the start of the experiment, crabs were kept in water to minimize stress and carefully cleaned of all decorations under a dissecting microscope using tweezers and a probe. Caution was taken to minimize damage to setae. Animals were then patted dry using Kim wipes, measured for carapace width and length using digital calipers, and weighed on a balance (RADWAG PS 3500/C/2, RADWAG, Radom, Poland). This procedure did not induce noticeable stress in *P*. *tumida*, which is characterized by overturning and difficulty with righting (personal observation).

Within each experimental treatment (ambient and reduced pH), 12 crabs were allowed to decorate (decorated crabs) while the other 12 crabs were not allowed to decorate (undecorated crabs). Decorations were provided each week only to the decorated crabs in each treatment. *Pelia tumida* primarily uses sponge for decoration, but it also covers with red algae, bryozoans, and hydroids (pers. observ.). As most animals were thoroughly covered in sponge at the time of collection, this was the decoration chosen for the experiment. Two sponge species, *Halichondria panacea* and *Haliclona permollis*, were collected from the Scripps Pier flume and held in a separate flow-through tank. Preliminary observation revealed that crabs showed no preference between the two sponge species. New sponge cut to 3 cm × 3 cm × 1 cm pieces were given to each decorated crab once per week so that they could decorate *ad libitum* for 24 hours, after which time the sponge was removed. Decorator crabs typically decorate immediately once a decoration source is provided, so this time was considered sufficient for crabs to decorate^49,50^.

**Figure 1:**
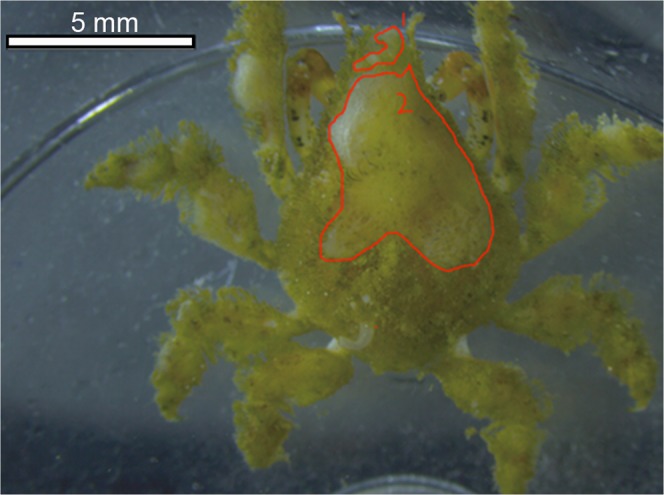
Decorated *Pelia tumida* under a dissecting microscope. Red outlines encompass the area of sponge used to calculate the percent cover of sponge for each animal. Scale bar = 5 mm.

Weekly photographs were taken of the dorsal carapace of each crab using an Iphone 6 camera to track decoration throughout the duration of the experiment. To minimize disturbance to the animals, crabs were kept in seawater while being transferred to a small container for imaging. A small grid was included in the image for calibration. Both carapace area and total area of sponge (including sponge extending beyond the carapace) were traced and calculated using ImageJ software (Fig. 1). Percent cover was calculated as total area of sponge divided by carapace area. Though some decoration is placed on the legs, it was minimal compared to the decoration on the carapace; thus only carapace decoration was evaluated. To equalize handling stress among treatments, undecorated crabs were sham-handled each time that the decorated crabs were photographed, using the same protocol, but with no photograph taken. At the end of the experiment, final decoration percent cover was quantified by photographing the dorsal carapace of each crab using a HD digital camera (Leica DFC290, Buffalo Grove, 206 Illinois, United States) attached to a stereomicroscope (Leica M165 C, Buffalo Grove, Illinois, United States). From these images, percent sponge cover was measured and calculated using the same method as described for the weekly measurements.

Immediately after imaging, decorations were carefully removed from each crab, dried in a fume hood for 6 days, and then weighed using a digital microbalance (Sartorius 1602 MP 6, Data Weighing Systems, Inc., Elk Grove, Illinois, United States). Decoration behavior was characterized as both the mass of sponge carried and the percent cover of sponge.

Once cleaned, crabs were measured and weighed by the procedure described above. The crabs were then euthanized by being placed in a −20 °C freezer for 30 minutes before being dissected for analyses of exoskeleton morphology and organic content. None of the crab dissections revealed evidence of apolysis, the earliest sign of premolt, so all crabs used for analysis were considered to be in the intermolt stage.

### Exoskeleton calcification

Cleaned crab carapaces were bisected so that one half could be analyzed for structure and elemental content using scanning electron microscopy (SEM) and the other half of the carapace analyzed for elemental composition using inductively coupled plasma mass spectroscopy (ICP-MS).

For SEM, carapace samples were fractured at a consistent location in the mesobranchial region using forceps, and then dried in a critical point drier (AutoSamdri 815 Series A, Tousimis, Rockville, Maryland, United States), secured to a double 90° SEM mount revealing the cross-section, and sputter coated with iridium. Cross-sections of carapace samples were then examined with an ultra-high resolution SEM equipped with EDX (XL30 SFEG with Sirion column, Field Emission Incorporated, Hillsboro, Oregon, United States). SEM imaging was done at 10 kV acceleration voltage. An overview image of the whole cuticle cross-section along with magnified images of the epicuticle, exocuticle and endocuticle layers were taken for each animal. Total cuticle thickness comprised all three visible layers and was averaged from 5 measurements taken from each whole cuticle image.

Mineral composition of the carapace cuticle was examined using EDX by magnifying each image so that the whole cuticle filled the screen. Spectra were collected at a 20 kV acceleration voltage and a minimum of 5,000 counts per second. A semi-quantitative analysis of all elements in the cuticle cross-section was conducted on all samples. Ca and Mg, which are key elements in cuticle mineralization, along with C, O, Na, Cl, Al, P, and S were found consistently in all samples, with some samples also containing small amounts of Si and K. We focused specifically on the amount of Ca and Mg in each sample, which was calculated as the weight percent (wt.%) relative to all detected elements, excluding the iridium coating.

For elemental trace analysis using ICP-MS, cleaned carapace samples were weighed and placed in Teflon vials for digestion with 0.5 mL of concentrated Teflon-distilled (TD) nitric acid (HNO_3_) on a hotplate at 120 °C for >24 h. Samples were dried and diluted by a factor of 4000 with 2% TD HNO_3_ before being transferred to pre-cleaned centrifuge tubes for analysis. Samples were doped with an indium solution at this time to monitor instrumental drift. Measurements were done using a *ThermoScientific iCAPq* c ICP-MS (Thermo Fisher Scientific GmbH, Bremen, Germany) in standard mode. Masses of Mg and Ca were sequentially measured for 30 ratios, resulting in internal precision of <2% (2 s.d.). Elements were corrected for total mole fraction (ambient pH/decorated *n* = 10, ambient pH/undecorated *n* = 8, reduced pH/decorated *n* = 10, reduced pH/undecorated *n* = 9). Raw data were corrected off line for instrument background and drift. Samples were bracketed by internal standards of crab carapace (*n* = 2), which allowed for calculations of absolute values. The standards yielded external precision of better than 1% for Mg and Ca (2 s.d.).

### Organic content

The metabolic state of an organism can be inferred through its overall organic content^32,51,52^. Here, we refer to organic content as the overall mass of proteins, lipids and carbohydrates. A crab with sufficient energy resources presumably has relatively higher organic content, and thus higher lipid stores and protein, compared to animals with higher energetic costs, which require the burning of this organic content to supply energy. Therefore, we designate animals with lower organic content as having higher energetic costs and higher metabolism^32^. Organic content is thus considered a proxy for metabolic activity.

After removal of the carapace, all internal organs and soft tissues, including the hepatopancreas (the primary storage organ), were carefully removed using a probe and forceps to excise all tissue from the cephalothorax. Due to the small size and improbability of separating each organ, all internal tissues were massed together for analysis. To prevent degradation of tissue, samples were placed in a sealed petri dish and stored in a −80 °C freezer for up to 50 days until analysis was performed.

**Figure 2:**
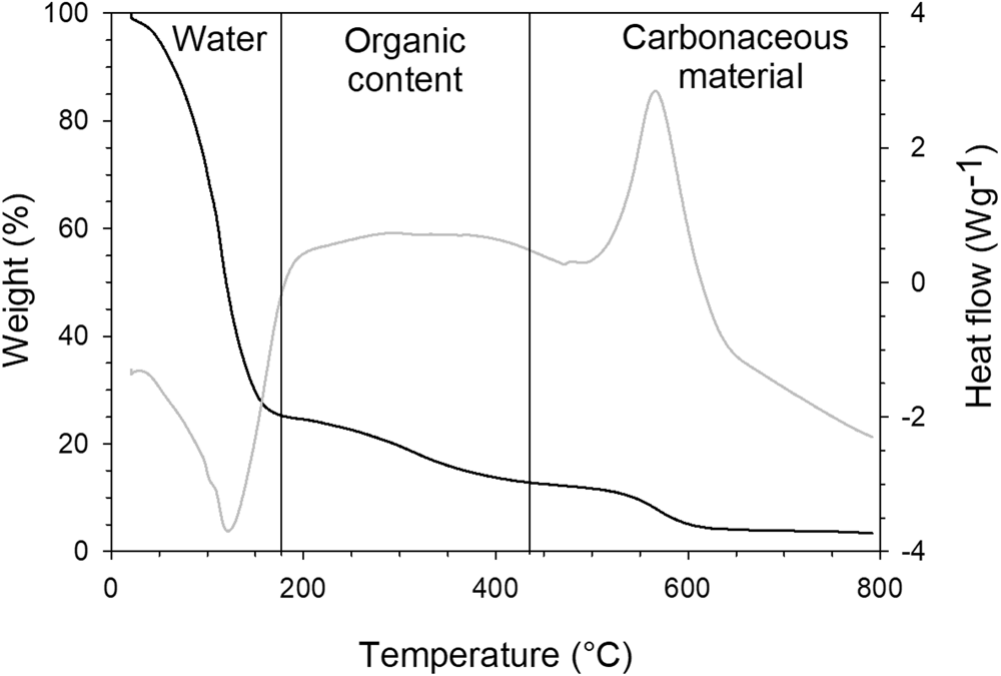
Example of TGA curve for *Pelia tumida* in ambient pH/decorated treatment. The black curve represents weight percent of sample, which decreases as the tissue is burned during pyrolysis. Changes in slope reflect changes in heat flow, represented by the gray curve, indicating a change in the type of material being burned. The first vertical black line marks the water burn-off point (~200 °C), indicating the removal of all water from the tissue. The second vertical black line (450 °C) marks the temperature for which organic material is removed from the tissue. The remaining material is carbonaceous.

Organic content of each crab was attained through thermogravimetric analysis (TGA) of the tissue sample using a SDT Q600 (TA Instruments, New Castle, Delaware, United States). Samples were heated in a 9 μL alumina crucible, from room temperature to 800 °C, at a rate of 10 °C/min in air. Previous studies using TGA on biological samples aided in interpretation and temperature designations of our TGA curves^53–55^. Within our sample curves, water was observed to burn off at approximately 200 °C, proteins, lipids and carbohydrates burned from 200 °C to 450 °C, and carbonaceous materials burned from 450 °C to the end of the procedure (Fig. 2). The temperature of 450 °C was chosen as the end point of the organic content burn off because it was consistent with temperatures in previous studies on microalgae (476 °C^54^), and it was invariable throughout all samples. Organic content was estimated by measuring the loss of tissue mass between 180 °C and 420 °C. Percent water content was also calculated because studies have shown that changes in water content are inversely related to changes in organic content, so we would therefore expect an increase in water content as metabolism increases and organic content decreases^56^. The use of TGA ensures that no water or carbonaceous materials impacted the determination of organic content. We chose to measure tissue pyrolysis instead of respirometry as a proxy for metabolism primarily because of complications with using live decoration that also consumes oxygen, as noted by Berke and Woodin^32^. Caloric intake was not used due to difficulty in collecting and measuring the debris of unconsumed food.

### Statistical analyses

All data were tested for normality using a Shapiro-Wilk test and for homogeneity of variance using Levene’s test. Decoration, change in mass, mineralization, and organic content were compared across treatments using two-way ANCOVAs with initial crab mass as a covariate. Sex was included as a factor because both males and females were used in the experiment. Mineralization data (wt.% Ca, wt.% Mg, Ca, Mg) were log transformed prior to analysis. Statistical analyses were performed in R Version 3.2.3. All data are reported as mean ± standard deviation.

## Results

### Water chemistry

Experimental pH was stable throughout the experiment for both ambient (8.01 ± 0.03) and reduced (7.74 ± 0.02) pH treatments. Temperature also remained consistent and the same among treatments throughout the experiment (ambient pH: 21.8 ± 1.2 °C; reduced pH: 21.8 ± 1.2 °C) (Mann-Whitney U test, *U* = 577632.0, *N*1 = 1084, *N*2 = 1102, *P* = 0.18), except for the last three days over which the temperature steadily decreased to 17.4 °C in both treatments.

### Survival and mass

**Figure 3:**
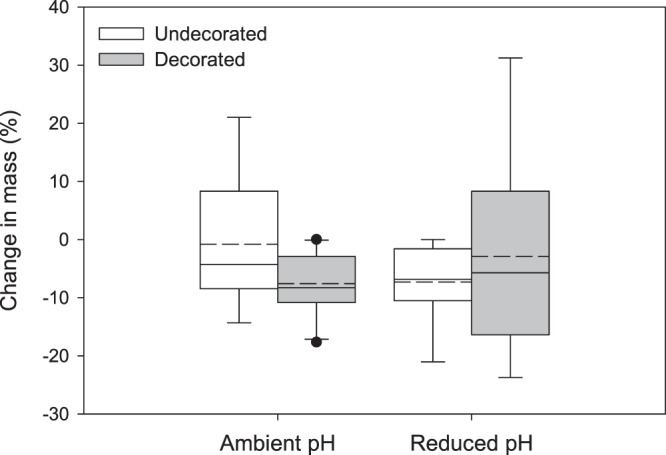
Percent change in mass for decorated and undecorated crabs in ambient and reduced pH treatments. Box boundaries = 25^th^ and 75^th^ percentiles, error bars = 90^th^ and 10^th^ percentiles, solid line = median, dashed line = mean. Outliers, represented by black dots, were included in the analyses.

Similar levels of mortality occurred among treatments, with 4 deaths in ambient pH/undecorated (3 female, 1 male), 2 in ambient pH/decorated (both female), 3 in reduced pH/undecorated (all male), and 3 in the reduced pH/decorated (all male). These deaths occurred randomly over the duration of the experiment and could not be attributed to any specific factor. We do not expect that fatality was due to handling during decoration removal because of the time course of deaths and because the procedure had no effect on crabs during preliminary work. It is possible that limited diet breadth or feeding frequency may have caused inadvertent stress, which is supported by the loss in mass across treatments, as described below. The experiment was ended after five weeks to prevent further mortality. During this time, only 2 animals molted (one from each of the undecorated treatments) and were excluded from analyses. This left sample sizes of N = 9 ambient pH/decorated, N = 8 ambient pH/undecorated, N = 9 reduced pH/decorated, and N = 8 reduced pH/undecorated for all analyses. Growth associated with molting was not assessed in this experiment, but intermolt changes in mass were. Most crabs experienced a loss in mass (ambient pH/decorated: −7.54 ± 5.54%; ambient pH/undecorated: −0.77 ± 11.80%; reduced pH/decorated: −2.87 ± 17.46%; reduced pH/undecorated: −7.27 ± 6.53%) (Fig. 3). The percent change in mass was highly variable among individuals, but did not differ among treatments (two-way ANCOVA: initial crab mass = covariate, *F* (3,24) = 1.19, *P* = 0.34), and there was no effect of initial crab body mass (*F* = 2.58, *P* = 0.12), but female crabs lost more mass (*F* = 4.70, *P* = 0.04). The variance in percent change in mass did not differ among treatments (Levene’s test, *F* = 2.13, *P* = 0.12).

### Exoskeleton structure and composition

**Figure 4:**
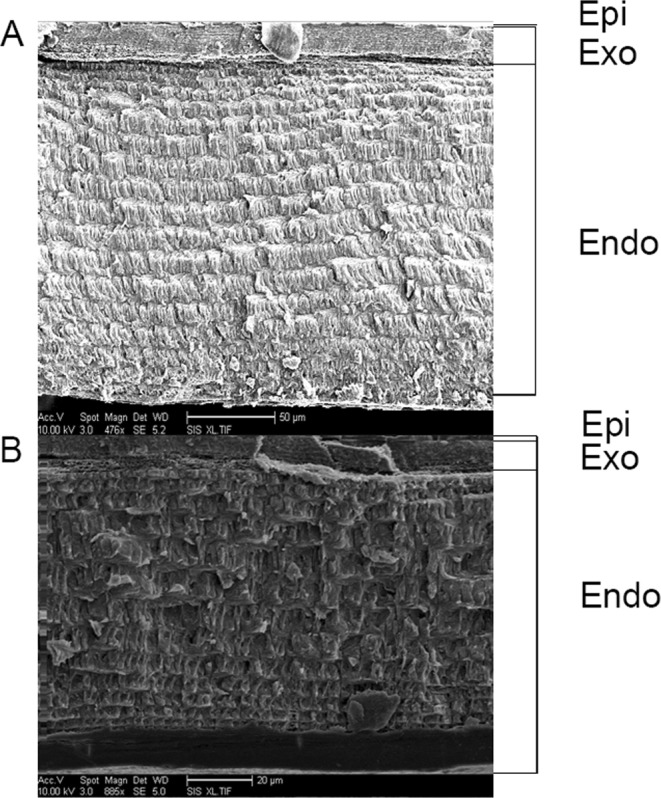
Representative scanning electron micrographs of carapace cuticle cross-section from undecorated crabs. Cuticle layers are noted on the right, epi = epicuticle, exo = exocuticle, endo = endocuticle. Scale bar for (**A**) = 50 μm and (**B**) = 20 μm.

There were no visible differences in the structure (e.g., layer thickness) of the carapace cuticle between treatments (Fig. 4). Total cuticle thickness of the carapace did not differ between pH or decoration treatments (ambient pH/decorated: 0.014 ± 0.004 mm; ambient pH/undecorated: 0.013 ± 0.003 mm; reduced pH/decorated: 0.012 ± 0.001 mm; reduced pH/undecorated: 0.012 ± 0.002 mm) (two-way ANCOVA, initial crab mass = covariate, *F*(3,23) = 1.91, *P* = 0.16), with no effect of sex (*F* = 3.41, *P* = 0.08) or interaction between sex and treatment (*F* = 0.77, *P* = 0.52).

**Figure 5:**
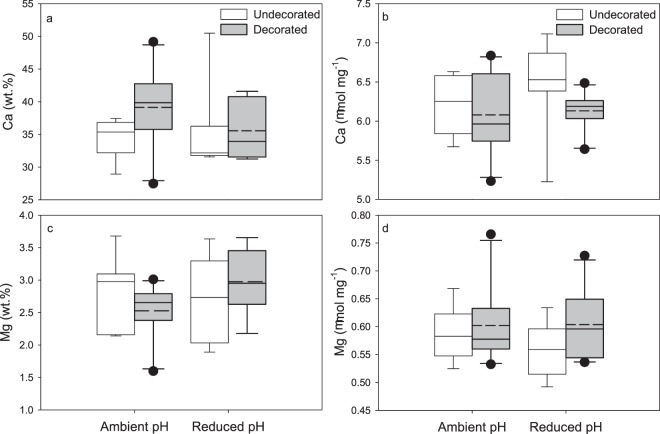
Mineral content of carapace cuticle. (**a**) Relative percent Ca determined by EDX. (**b**) Total Ca content determined by ICP-MS. (**c**) Relative percent Mg. (**d**) Total Mg content. Box plot boundaries = the 25^th^ and 75^th^ percentiles, error bars = 90^th^ and 10^th^ percentiles, solid line = median, dashed line = mean. Outliers, represented by black dots, were included in the analyses.

Elemental analysis of the carapace cuticle with EDX showed no significant difference in wt.% Ca (ambient pH/decorated: 39.15 ± 6.1%; ambient pH/ undecorated: 34.25 ± 3.1%; reduced pH/decorated: 35.57 ± 4.5%; reduced pH/undecorated: 34.97 ± 6.1%) among pH and decoration treatments (two-way ANCOVA, initial crab mass = covariate, *F*(3,24) = 1.82, *P* = 0.17), with an effect of sex (*F* = 7.22, *P* = 0.01) but no interaction between sex and treatment (*F* = 0.73, *P* = 0.55) (Fig. 5a). ICP-MS analysis of the mean concentration of Ca in the carapace cuticle confirmed the EDX results, showing no difference among treatments (ambient pH/decorated: 6.08 ± 0.50 μmol mg^−1^; ambient pH/ undecorated: 6.22 ± 0.37 μmol mg^−1^; reduced pH/decorated: 6.13 ± 0.25 μmol mg^−1^; reduced pH/undecorated: 6.49 ± 0.54 μmol mg^−1^) (two-way ANCOVA, initial crab mass = covariate, *F*(3,23) = 1.00, *P* = 0.41), with no effect of sex (*F* = 0.40, *P* = 0.53) or interaction between sex and treatment (*F* = 1.15, *P* = 0.35) (Fig. 5b). There was also no difference in wt.% Mg among treatments (ambient pH/decorated: 2.53 ± 0.4%; ambient pH/ undecorated: 2.83 ± 0.5%; reduced pH/decorated: 2.98 ± 0.5%; reduced pH/undecorated: 2.70 ± 0.6%) (two-way ANCOVA, initial crab mass = covariate, *F*(3,24) = 1.10, *P* = 0.37), also with no effect of sex (*F* = 0.78, *P* = 0.39) or interaction between sex and treatment (*F* = 0.27, *P* = 0.85) (Fig. 5c). Likewise, the mean concentration of Mg did not differ among treatments (ambient pH/decorated: 0.60 ± 0.07 μmol mg^−1^; ambient pH/ undecorated: 0.59 ± 0.05 μmol mg^−1^; reduced pH/decorated: 0.60 ± 0.06 μmol mg^−1^; reduced pH/undecorated: 0.56 ± 0.05 μmol mg^−1^) (two-way ANCOVA, initial crab mass = covariate, *F*(3,23) = 1.04, *P* = 0.39), with no effect of sex (*F* = 1.22, *P* = 0.28) or interaction between sex and treatment (*F* = 0.71, *P* = 0.56) (Fig. 5d).

### Organic content

**Figure 6:**
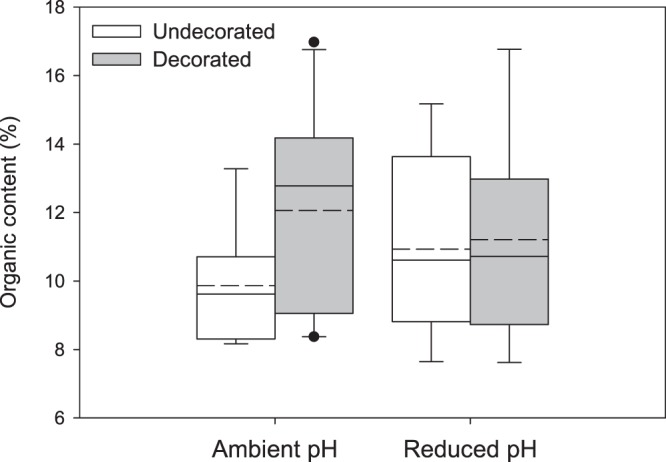
Mean percent organic content of crabs from each treatment. Organic content includes lipids, carbohydrates and proteins and is calculated from TGA curves. Box plot boundaries = the 25^th^ and 75^th^ percentiles, error bars = 90^th^ and 10^th^ percentiles, solid line = median, dashed line = mean. Outliers, represented by black dots, were included in the analyses

Percent total organic content of crab soft tissue did not differ between pH or decoration treatments (ambient pH/decorated: 12.05 ± 3.0%; ambient pH/undecorated: 9.86 ± 1.7%; reduced pH/decorated: 11.21 ± 3.0%; reduced pH/undecorated: 10.93 ± 2.7%) (two-way ANCOVA, initial crab mass = covariate, *F*(3,24) = 0.62, *P* = 0.61), with no effect of sex (*F* = 1.74, *P* = 0.20) or interaction between sex and treatment (*F* = 0.44, *P* = 0.72) (Fig. 6). The percent of total water content supported this result, also showing no significant differences between pH and decoration treatments (ambient pH/decorated: 24.03 ± 4.4%; ambient pH/undecorated: 21.76 ± 3.4%; reduced pH/decorated: 23.32 ± 4.8%; reduced pH/undecorated: 22.43 ± 4.3%) (two-way ANCOVA, initial crab mass = covariate, *F*(3,23) = 1.04, *P* = 0.39), with slightly more water content in females (*F* = 5.37, *P* = 0.03) and no interaction between sex and treatment (*F* = 0.25, *P* = 0.86).

### Decoration behavior

**Figure 7:**
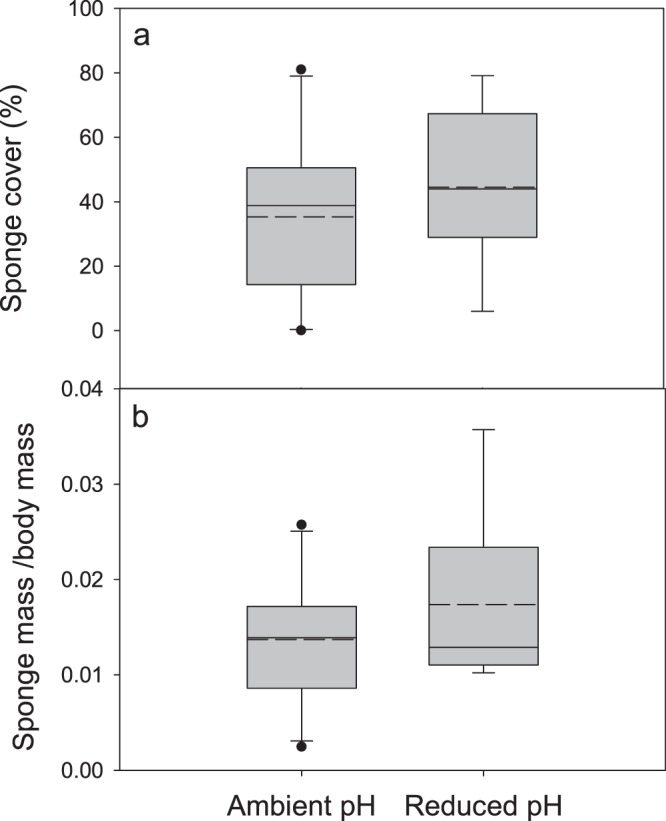
Sponge decoration. (**a**) percent sponge cover and (**b**) sponge mass (g) divided by body mass (g) (to correct for body size) from crabs in ambient and reduced pH treatments. Box plot boundaries = the 25^th^ and 75^th^ percentiles, error bars = 90^th^ and 10^th^ percentiles, solid line = median, dashed line = mean. Outliers, represented by black dots, were included in the analyses.

Neither measure of decoration behavior (sponge percent cover and mass) differed among treatments. Percent sponge cover was the same for crabs in both pH treatments throughout the duration of the experiment (Fig. 7a) (two-way repeated measures ANCOVA: initial crab mass = covariate, *F*(1,95) = 1.69, *P* = 0.20), with an effect of week (*P* < 0.05), but no interaction effects of week, sex or mass with treatment (*P* = 0.84, 0.42, 0.88, respectively). Crabs from both pH treatments had significantly less percent cover at week one (Holm-Sidak: all *P* < 0.05 for week one versus weeks 2–5) than the remainder of the experiment, for which there were no differences between weeks (Holm-Sidak: all *P* > 0.05 for weeks 2–5). The mean mass of sponge carried by individuals at the end of the experiment was not significantly different between pH treatments when controlled for initial crab body mass (two-way ANCOVA: (1,13) = 4.62, *P* = 0.71), and there was no effect of sex (*F* = 0.87, *P* = 0.37) or interaction between treatment and sex (*F* = 0.51, *P* = 0.49), but there was an effect of initial crab mass (*F* = 13.05, *P* < 0.005). Variance in sponge mass did not differ among treatments (Levene’s test: *F* = 1.05, *P* = 0.39). Size-corrected sponge cover (sponge mass divided by initial crab mass) was 0.012 ± 0.006 in ambient pH and 0.017 ± 0.009 in reduced pH (Fig. 7b). Though the undecorated crabs were not given sponge to decorate with, some individuals had small amounts of sponge on their carapace at the end of the experiment [ambient pH: 0.0014 ± 0.0025 g (*n* = 2), reduced pH: 0.0031 ± 0.0035 g (*n* = 5)].

## Discussion

The decorator crab *Pelia tumida* shows tolerance to pH levels that mimic forecasted near-term changes in ocean chemistry (decrease 0.3–0.4 in pH by 2100^43^). Such experimental conditions are sufficient to elicit a variety of detectable responses in other crustacean species, including changes in survival, metabolism, and calcification^1,6,57^, but not for any of the variables measured in this study on *P*. *tumida*. While vulnerabilities may manifest in other biological aspects, *P*. *tumida* appears to be resilient to moderate pH reductions, at least over the time-scale of this experiment. Length of exposure to reduced pH conditions is an important factor because some species take as long as a year to reveal measurable effects^58^, while others do so in as little as 3 weeks^11^. The natural environmental variability within this species’ range may factor heavily in their resilience, as coastal waters off California are thought to be naturally stressful because of changes in temperature and calcite saturation states that result from upwelling events^59^. The experimental pH used in this study on *P*. *tumida* was well below what they experience in the subtidal habitat they were collected from, which ranges from 7.9 to 8.13 (based on data logged every 15 min for 16 consecutive months; data from Kram, S. L., Takeshita, Y., Dickson, A., Martz, T. & Smith, J. E. Scripps Ocean Acidification Real-time [SOAR] Dataset, Scripps Institution of Oceanography). Yet, inhabiting a variable pH environment does not necessarily confer resilience. Forecasted changes in ocean pH may overextend the physiological tolerance limits of near-shore animals, resulting in detectable responses. This is evidenced in metabolic and thermal tolerance changes of intertidal porcelain crabs^20,60^ and changes in growth and calcification of intertidal barnacles^13,61^. *Pelia tumida* likely has a broad physiological tolerance range similar to that of mantis shrimp, which experience no stress at pH values far below what they encounter in nature^15^. This is surprising given that *P*. *tumida* is a relatively inactive, slow-moving, osmoconforming species, which are traits thought to reduce the capacity of animals to respond to changes in external pH^1,17,18^.

Calcification is a critical metric in evaluating the response of crustaceans to ocean acidification conditions, and our conclusion that this process is unaffected in *P*. *tumida* is limited to intermolt maintenance processes. Examples of increased calcification in crustaceans responding to reduced pH conditions occurs during the molting process, because calcification peaks immediately following ecdysis, when new exoskeleton is formed and hardened through cross-linking and deposition of calcium carbonate. Too few crabs molted in this experiment to evaluate molt-related calcification. Albeit small compared to post-molt calcification, there is a flux of calcium across the epithelium and a net uptake of calcium in the exoskeleton that takes place during the intermolt stage^62–64^. This was recently observed in intermolt mantis shrimp, where calcium content of the cuticle continued to increase up to 6 months following molting, indicating long-term accretion^15^. More significantly, we did not detect any evidence that dissolution of the exoskeleton occurred, as might be expected in osmoconformers if their hemolymph pH decreases. Neither Ca nor Mg quantities of the exoskeleton differed under reduced pH conditions, indicating that these elements remained in the exoskeleton rather than accruing in the hemolymph as a consequence of dissolution. There appears to be no metabolic effects related to the calcification process in *P*. *tumida*. While the calcification regulatory processes were unaffected by reduced pH conditions over the course of 5 weeks, it remains a possibility that effects may emerge over a longer exposure time.

Furthermore, like most crustacean ocean acidification studies that examine calcification, we focused only on one region of the exoskeleton. Due to the small size of *P*. *tumida*, we only sampled the carapace, but it is possible that other regions of the crab exoskeleton are more sensitive to reduced pH conditions, such as the chelae or setae. In mantis shrimp, for example, changes in Mg content occurred in the merus of the raptorial appendage, but not the carapace, under reduced pH^15^. This region-specific response to reduced pH was also observed in the velvet crab, *Necora puber*, which had increased Mg in the chelae, but not the carapace^14^. This variable response within an individual is not surprising given that the calcification process itself is malleable, enabling localized control of mineral content for regions that support specialized functions. Inherent variation in calcification within an individual animal requires that localized measurements of mineral content from multiple exoskeleton regions be considered if accurate descriptions of the crustacean response to environmental stressors are to be made.

Maintaining homeostasis of physiological processes under environmental stress requires additional energy for some organisms, materializing as increased metabolism and reduced growth rates. This energetic trade-off has been observed in a variety of marine calcifiers whose strong acid-base regulation maintains calcification under reduced pH conditions, but at metabolic expense^6,10,13^. For instance, when exposed to moderate increases in pCO_2_ [991 µatm^65^ and 490 µatm, 1,100 µatm, 2,400 µatm^66^], another decorating majid crab, *Hyas araneus*, sustained net calcification, but experienced increased metabolism and stress. We predicted a similar response in the decorator crab *P*. *tumida*, but it showed no indication of metabolic costs, as measured by organic content, under reduced pH conditions. The current study did not, however, take into account the complexity of a species’ metabolic response to environmental pH. When *H*. *araneus* was exposed to a high pCO_2_ level of 1,960 µatm, metabolic rate decreased rather than increased^65^, revealing a disparity of outcomes specific to carbon chemistry parameters. Reduced metabolic rates under increased pCO_2_ conditions have been observed in other crustaceans as well, including the velvet swimming crab, *Necora puber*^14^, and the prawn, *Metapenaeus joyneri*^26^. Metabolic depression is thus another potential outcome for animals exposed to environmental stress, but it is typically associated with reduced growth rates^18,67,68^. *Pelia tumida* showed no signs of elevated or depressed metabolism, based on organic content, suggesting that the pH conditions used in this experiment either did not impose a sufficient stress to affect metabolism or that the crab’s energy budget already accounts for physiological energetics under stress^22^.

Surprisingly, we did not detect an energetic cost of decorating for *P*. *tumida*, in contradiction to expectation and previous studies on decoration behavior energetics in decorator crabs and sea urchins^29,32^. The cost of decoration behavior has only been quantified in one species of decorator crab, and is likely inconstant given the diversity of species and the interconnection of habitat, body size, ontogeny and sexual dimorphism with the evolution of decorating in this group. The energetics required for decorating are presumably unique to the individual species. Decorator crab species that do not undergo an ontogenetic shift in decoration behavior, such as *P*. *tumida*, tend to be small (5–40 mm carapace width^32^), presenting the possibility that decorating may not be as costly for smaller crabs. Yet, *Oregonia gracilis* demonstrates a cost of decoration for both juveniles (3–6 mm) and subadults (11–18 mm)^32^, refuting the idea of a size refuge from decoration costs. Species may minimize decoration costs by using different types and amounts of decoration material or by reducing activity and being slow-moving^69^. Indeed, small decorator crabs facilitate blending in with their background by remaining still and inactive during daylight hours^28^.

Our main hypothesis was that under energetically stressful conditions, decoration behavior would be reduced to allocate energy to other important physiological processes, as was observed in the sea urchin *Strongylocentrotus droebachiensis*^29^. However, *P*. *tumida* did not change its decoration behavior (percent cover and total mass of sponge) when maintained under reduced pH conditions. This outcome is sensible because there were no indications of energy limitation related to pH conditions; both change in crab mass (wet weight) and organic content were the same across treatments. Reduced pH alone does not appear to induce sufficient sub-lethal stress to affect metabolism or behavior in *P*. *tumida*. However, if ocean acidification is considered in concert with other climate change stressors, such as increasing temperature, declining oxygen, or changes in salinity, the interactive effects may induce measureable stress in *P*. *tumida*.

Typically studies of energetic trade-offs carefully control and monitor energy intake^70,71^, which is why studies on decoration behavior energetics often use starvation conditions^32^. We chose not to introduce starvation stress in this experiment in an attempt to explore realistic responses to reduced pH conditions. The natural energy intake of *Pelia tumida* is unknown, but they inhabit areas with abundant algae and invertebrates and are likely not food-limited in nature. In this study, crabs were fed to satiation three times per week, but either this amount was not adequate or the singular diet did not provide complete nutrition because almost all crabs experienced a small loss in mass by the end of the experiment. The consistency suggests that it is more than measurement error in wet weights. In spite of crabs being food limited, pH conditions had no effect on metabolism, calcification, and decoration behavior. It is also germane that while we fed crabs consistent, measured amounts of food throughout the experiment, quantifying unconsumed food was not feasible. We therefore have no precise measure of the amount of food consumed per individual. If decorated animals in the reduced pH treatment had higher energetic costs, but satiated this with increased feeding, it was not detectable. Additionally, some species of decorator crabs are known to use their decorations as a food cache^72^. Percent sponge cover in individuals was not stable over the course of the experiment, indicating that some sponge may have fallen off, deteriorated, or possibly been consumed. Thus, some crabs may have consumed more food than others, providing them with higher caloric intake or different food quality. The variation in decoration material over time was the same for crabs in ambient and reduced pH treatments, thereby dispelling the possibility that decoration food cache was used to supplement energy in response to pH stress. Overall, metabolic costs may not have been detected because the decorator crabs were not energy limited in this experiment, an important factor for some species in coping with ocean acidification conditions^73^.

## Conclusions

Crustaceans exhibit responses to experimental ocean acidification conditions that vary among species, life histories, habitats, and experimental conditions, making it opportune, yet challenging, to decipher the critical aspects driving tolerance in this diverse group of animals. The current study on *P*. *tumida* revealed no changes at the organismal level, in terms of physiology (organic content; lipids, carbohydrates and proteins), morphology (Ca and Mg content), or behavior (decoration percent cover and mass), under reduced pH conditions. This species of decorator crab is able to maintain multiple biological processes while responding to decreases in external pH, which contrasts with other studies focusing on energy limitation and behavioral aspects in response to reduced pH and emphasizes the need for further crustacean research to include more species and a wider view of possible ecological responses.

## Data Availability

The datasets generated and analyzed during the current study are available from the corresponding author on request.
